# Oral health status and subgingival microbiota in children with juvenile idiopathic arthritis

**DOI:** 10.3389/fcimb.2026.1831655

**Published:** 2026-06-26

**Authors:** Ceyhun Açarı, Yavuz Oktay, Gülser Kılınç, Leman Binokay, Serkan Türkuçar, Hatice Adıgüzel Dundar, Tutku Yaraş, Pelin Teke Kısa, Gökhan Karakülah, Nur Arslan, Mehmet Ali Öktem, Şevket Erbil Ünsal

**Affiliations:** 1 Pediatric Rheumatology, Inonu University, Malatya, Türkiye; 2 Izmir Biomedicine and Genom Center-IBG, Izmir, Türkiye; 3 Department of Children’s Dentistry, Dokuz Eylul University, Izmir, Türkiye; 4 Pediatric Rheumatology, Pamukkale University, Denizli, Türkiye; 5 Pediatric Rheumatology, Behcet Uz Children’s Hospital, Izmir, Türkiye; 6 Pediatric Metabolism and Nutrition, Dokuz Eylul University, Izmir, Türkiye; 7 Department of Microbiology, Dokuz Eylul University, Izmir, Türkiye; 8 Pediatric Rheumatology, Dokuz Eylul University, Izmir, Türkiye

**Keywords:** juvenile idiopathic arthritis, oral health, oral microbiota, peptostreptococcus, periodontitis, porphyromonas

## Abstract

**Objective:**

This study aimed to compare the oral and dental health status and the oral microbiota of patients with juvenile idiopathic arthritis (JIA) with those of healthy children.

**Methods:**

The 60 patients with JIA and 26 healthy children were included in the study. Decayed, missing, filled teeth index (DMFT/dmft), oral hygiene index (OHI), gingival index (GI), and papillary bleeding (PB) index analyses were performed for the permanent and deciduous teeth of the patients in the pediatric dental clinic. Subgingival plaque samples were taken from the gingival groove and tooth surface with a sterile swab and stored in a -80C freezer. Following the DNA isolation, the analysis of microbiota content was performed by 16S rRNA gene sequencing method.

**Results:**

The oral hygiene index (p=0.045), debris index (p=0.000), gingival index (p=0.001), and papillary bleeding index (p=0.002) were significantly higher in the patient group than in the control group. Oral microbiome analysis found no significant difference in alpha and beta diversity between the groups. *Porphyromonas* and *Peptostreptococcus* (Peptostreptococcales-Tissierellales) were more common in JIA, while the presence of *Atopobium*, *Scardovia*, *Rothia*, and *Propionivibrio* were higher in the control group.

**Conclusions:**

In our study, The oral hygiene index (OHI) value, debris index (DI), and PBI were significantly higher in the patient group compared to the control group. Some bacteria such as *Porphyromonas* and *Peptostreptococcus* were more common in the JIA group, compared to the control group. This study shows that the oral health and oral microbiota populations of children with JIA may be altered compared to healthy children. These changes may create a predisposition to local inflammatory processes and potentially contribute to the onset and severity of the disease.

## Highlights

- Oral microbiota profiles may be altered in children with JIA compared to healthy children.
- Oral microbiota changes may predispose to inflammatory processes
- Dysbiosis may contribute to the onset and severity of the disease.

## Introduction

Humans have co-evolved with microorganisms that play a vital role in the immune system and homeostasis (Vieira et al., 2014). The term microbiota, defined in the early 2000s, refers to the sum of commensal and pathogenic microorganisms, their genomes, and environmental interactions in a particular biological region (Lederberg and McCray, 2001). Oral and gut microbiotas, in particular, stand out as the most influential ecosystems in shaping the systemic immune response. Disruption of the balance between these complex microbial communities in the oral cavity and intestinal system and the immune system plays a critical role in the pathogenesis of adult-onset chronic inflammatory diseases, including rheumatoid arthritis (RA) (Rosenstein et al., 2004; Scher et al., 2012).The oral cavity represents a highly complex microbial ecosystem composed of diverse microorganisms inhabiting mucosal surfaces, subgingival plaque, and saliva. Gingivitis and the subsequent development of periodontitis are characterized by neutrophilic inflammation, matrix metalloproteinase activity, and the production of inflammatory cytokines that may ultimately lead to bone erosion (Lange et al., 2016). These pathological processes share notable similarities with the inflammatory mechanisms observed in the synovium and adjacent bone tissues in inflammatory arthritis. Consequently, a potential biological link between periodontal inflammation and systemic inflammatory arthritis has long been proposed.

Epidemiological evidence suggests a bidirectional relationship between periodontitis and rheumatoid arthritis (RA), with a higher prevalence of each condition observed in patients suffering from the other. The shared pathogenetic basis of these diseases is anchored in chronic inflammatory processes mediated by a dysregulated immune response and an overproduction of proinflammatory cytokines, notably tumor necrosis factor-alpha (TNF-α), interleukin-1 beta (IL-1β), and interleukin-6 (IL-6). These cytokines stimulate the production of matrix metalloproteinases (MMPs), which drive progressive tissue destruction, manifesting as periarticular bone erosion in RA and alveolar bone loss in periodontitis. Furthermore, a crucial molecular link has been identified in the process of protein citrullination. In RA, the loss of immune tolerance to citrullinated proteins is a hallmark of disease progression. Emerging evidence indicates that Porphyromonas gingivalis, a keystone pathogen in periodontitis, expresses its own peptidylarginine deiminase (PAD) enzymes. This pathogen-derived enzyme can facilitate the citrullination of host proteins, thereby triggering citrulline-specific autoimmunity and potentially initiating or exacerbating the underlying pathology of RA (Lundberg et al., 2010; Araujo et al., 2015; Berthelot and Le Goff, 2010; Erciyas et al., 2013).

Juvenile idiopathic arthritis (JIA) is the most common chronic rheumatic disease in childhood and arthritis that persists for at least 6 weeks is a common clinical finding (Makay et al., 2018). Research on the relationship between childhood chronic arthritis and the microbiome is very scarce, and a significant part of it is related to the gut microbiota. Di Paola et al. examined fecal microbiota and found that JIA subgroups had an increase in some bacterial groups (Ruminococcaceae) and a decrease in others (Clostridiaceae, Peptostreptococcaceae) compared to the control group (Di Paola et al., 2016). The relationship between oral microbiota and periodontal disease and JIA has recently become a point of interest. Evidence of *Porphyromonas gingivalis* has been found in many subtypes of JIA (Romero-Sanchez et al., 2017). Lange et al. stated that periodontitis may be a factor in the etiology of JIA due to higher antibody titers against *Porphyromonas gingivalis* and poorer oral hygiene in children with cyclic citrullinated peptide (CCP) - positive JIA (Lange et al., 2016).

Although the association between the oral environment and Juvenile Idiopathic Arthritis (JIA) has been previously explored, most existing studies have primarily relied on salivary samples to characterize the oral microbiota. However, saliva may not fully reflect the site-specific microbial dysbiosis occurring in the periodontal niche. The present study addresses this gap by performing a comprehensive microbiota analysis of subgingival plaque samples harvested directly from the gingival sulcus and tooth surfaces. By investigating this specific niche—the primary site of periodontal inflammation—our study provides a more precise and in-depth assessment of the microbial landscape in children with JIA. Consequently, this study aims to determine the oral health status and subgingival microbiota distribution of JIA patients and to compare these findings with those of a healthy control group.

## Materials and methods

The study included 60 Juvenile Idiopathic Arthritis (JIA) patients followed up at the Pediatric Rheumatology outpatient clinic of Dokuz Eylül University Faculty of Medicine. The cohort consisted of children diagnosed with polyarticular or enthesitis-related arthritis according to ILAR (International League of Associations for Rheumatology) classification criteria and who had a follow-up period of at least six months (Makay et al., 2018). Twenty-six children, who did not have any systemic diseases, and who had not used antibiotics in the last 12 weeks were included as a control group. Children with a history of dental or systemic disease, or those using antibiotics or oral antiseptic solutions were not included. Written consent was obtained from the children and their parents. The protocol was approved by the local ethical committee (2018/22-28) and complied with the declaration of Helsinki. The patient’s age, gender, age at diagnosis, follow-up period, and treatment regimens were recorded.

### Oral and dental evaluation

Oral examinations of the patients were performed by a specialist pediatric dentist in the pediatric dental clinic using a mirror probe under the light of a reflector. The dmft/DMFT (decayed, missing, filled teeth) index, which shows the number of decayed, lost, and filled teeth, was used in the evaluation of dental caries in permanent (DMFT) and primary teeth (dmft) in the mouth. dmft/DMFT index and oral hygiene index (OHI) were determined for the permanent teeth and deciduous teeth of the patients and recorded in the patient registration forms (World Health Organization, 2003). Greene’s recommended Simplified Oral Hygiene Index (OHI-S) was used to determine oral hygiene (Greene and Vermillion, 1964). This index includes debris (DI-S) and calculus index (CI-S). OHI-S is calculated on 6 threads [16^th^, 26^th^, 36^th^, 46^th^, 11^th^, and 31^st^ tooth]. For deciduous or mixed teeth, the last tooth of the arch and the deciduous incisors were evaluated. If there was no tooth in the recommended position, the tooth closest to it was evaluated. For each surface evaluated, the values of each component of DI-S and CI-S were added together and divided by the number of teeth.

The gingival index (GI), which was used to determine the presence of gingival inflammation, was performed by applying a periodontal probe along the buccal and lingual/palatal margins of all teeth. The data were evaluated on a scale of 1-3. The index was calculated by dividing the values of each tooth by the number of surfaces examined.

### Collection and preparation of samples

Prior to plaque collection, all participants were instructed to rinse their mouths with a saline solution to remove superficial debris. Subgingival plaque samples were collected using a standardized technique with a single-use sterile cotton-tipped swab. The collection protocol was strictly followed for all subjects to ensure consistency: plaque was gently scraped from the gingival sulcus and the buccal and palatal surfaces of the upper right and left first molars and maxillary incisors. Subsequently, the same sterile swab was used to collect samples from the gingival sulcus and the buccal and lingual surfaces of the lower right and left first molars and mandibular incisors. The entire sampling procedure was limited to approximately one minute to ensure uniformity across the cohort. Samples were immediately placed into sterile, capped microcentrifuge tubes without any transport medium and transferred on ice to the laboratory, where they were stored at −80 °C until DNA extraction.

### Microbiome DNA isolation and 16S NGS sequencing

The QIAamp DNA Microbiome Kit (Qiagen, #51704) was used for microbiome analysis. The collected swab samples were kept in 1 ml of PBS (phosphate buffered saline solution) and mixed for 30 sec, allowing the samples to pass from the cotton swab to the PBS. First, samples were incubated with the lysis buffer solution. Proteins and nucleic acids of host cells were cleaved using Proteinase K and proprietary guanidinium thiocyanate-based lysis buffer (Buffer ACL) included in the QIAamp DNA Microbiome Kit, respectively. The samples were then subjected to mechanical and chemical lysis by vortexing for 10 minutes in PowerBead tubes (2 ml screw-cap tubes pre-filled with 0.1 mm silica beads), a procedure that simultaneously disrupts bacterial cell walls through bead-beating and denaturing buffer activity, thereby releasing microbial DNA and microbial DNA to be released. These samples were then mixed with Buffer ACB (a chaotropic binding buffer that promotes nucleic acid adsorption to the silica membrane), loaded onto the column, and centrifuged. After the microbial DNA attached to the column was washed with a wash buffer, pure bacterial DNA was then eluted with an elution buffer. Quality and quantity measurements were made with a Nanodrop 2000 (Thermo) device. For further quality control of DNA, the V3-V4 region of the 16S rRNA gene was amplified by PCR using the primer pair 341F (5′-CCTACGGGNGGCWGCAG-3′) and 806R (5′-GGACTACHVGGGTWTCTAAT-3′) targeting the V3–V4 hypervariable region of the bacterial 16S rRNA gene from each sample and confirmed by agarose gel electrophoresis (Klindworth et al., 2013; Herlemann et al., 2011). A minimum of 5 ng/μl of purified DNA (as determined by Nanodrop 2000 spectrophotometry; A260/280 ratio between 1.8 and 2.0) was delivered to the service provider company Eurofins and libraries were prepared by adding adaptor sequences to the V3-V4 region amplified by PCR. After the quality determination with the Bioanalyzer 2100 (Agilent), libraries of appropriate quality were pooled and sequenced in the MiSeq (Illumina) device with a length of 2x300 cycles and an average of 100,000 reads/samples.

### Bioinformatics analysis of 16S rRNA gene amplicon sequencing data

Ampliseq v2.3.1 (https://nf-co.re/ampliseq) workflow, developed by nf-core (https://nf-co.re/) using Nextflow v21.10.6 (https://www.nextflow.io/), was used for bioinformatics analysis of the obtained sequencing data. Nextflow programming language allows bioinformatics analyses of next-generation sequencing data to be standardized and replicated independently of the platform used. Since reproducibility is one of the important factors in bioinformatics analysis, we used the nf-core/ampliseq workflow, which is known to provide reproducible results.

Because sequencing data originated from three independent MiSeq runs, the “--multiple_sequencing_run” flag was activated to enable run-specific error modelling in DADA2. Taxonomic classification was performed against the SILVA 16S rRNA reference database (version 138), specified via the “--dada_ref_taxonomy silva=138” parameter. A structured sample metadata table (containing sample identifiers, file paths, run identifiers, and group assignments) was prepared according to the nf-core/ampliseq documentation requirements and supplied as input to the workflow.

First, in the nf-core/ampliseq workflow, quality control of sequencing libraries was assessed using FastQC (v0.11.9) to evaluate per-base sequence quality and GC content across all samples. Low-quality bases and adapter/primer sequences were subsequently trimmed using Cutadapt (v3.4) with primer-specific parameters corresponding to the 341F/806R primer pair. After primer removal, the resulting high-quality reads were processed with DADA2 (v1.18), which performs the following sequential operations: (i) filtering and trimming of reads based on quality thresholds, (ii) estimation of sample-specific sequencing error rates, (iii) denoising of reads to infer true biological sequences, (iv) merging of paired-end reads (forward and reverse) into full-length amplicons, (v) removal of PhiX contamination (an internal sequencing control), and (vi) elimination of chimeric sequences generated during PCR amplification. The output of DADA2 consists of amplicon sequence variants (ASVs)—exact, single-nucleotide-resolution sequences that represent individual microbial taxa. Each ASV was assigned a taxonomy by comparing its sequence against the SILVA 16S rRNA reference database (version 138) using the DADA2 naive Bayesian classifier. In parallel, for operational taxonomic unit (OTU)-based analysis (in which sequences sharing ≥97% identity are clustered together), primer-trimmed sequences were additionally processed using QIIME2 (v2021.8) with default parameters. Alpha diversity (within-sample species richness and evenness, measured by observed OTUs, Shannon entropy, and Faith’s phylogenetic diversity) and beta diversity (between-sample compositional dissimilarity, assessed by Bray–Curtis and UniFrac distances) were calculated within the QIIME2 framework. A comprehensive quality control summary across all processing steps was compiled using MultiQC (v1.11).

Statistical analyses for the microbiome data were performed using R software version 3.6.0 (https://www.r-project.org/). Samples with a read number of 0 for at least 20% of bacterial genomes were excluded from subsequent analyses. Differential abundance analysis of bacterial taxa was performed using the EdgeR (v3.28.1) package, and P-values were calculated to determine statistically significant bacterial taxa between the patient and control groups. Data visualization operations were performed in the R environment with the help of the ggplot2 package.

On the other hand, the categorized clinical oral health parameters, including OHI, DMFT, DI, GI, and PBI scores, were analyzed as nominal/categorical variables and presented as frequencies with corresponding percentages [n (%)]. The cross-tabulated clinical data comparing the patient and healthy control cohorts were analyzed using Pearson’s Chi-square test. For contingency tables with low expected cell frequencies (<5) or zero values, Fisher’s exact test was utilized to determine statistical significance. All statistical computations for these clinical indices were performed using IBM SPSS Statistics software. For all analyses (both in R and SPSS), a two-tailed p-value < 0.05 was established as the threshold for statistical significance.

## Results

### Demographic and clinical oral health characteristics

A total of 60 JIA patients, 30 (50%) girls and 30 boys, were included in the study. The mean age of JIA patients was 12.7 ± 0.5 years. The age at diagnosis was 9.4 ± 0.6 years, and the follow-up period was 42.8 ± 5.2 months. Of the 26 healthy children included as the control group, 14 (53.8%) were female, and the mean age was 11.7 ± 0.8 years. There was no significant difference in the age distribution (p=0.294) and sex (p=0.747) between controls and patients. The JIA subtype distribution was 31 (51.7%) enthesitis-related arthritis and 29 (48.3%) polyartricular JIA.

The results of dental caries index values (DMFT+dmft), gingival index (GI), papillary bleeding index (PBI), and debris index (DI) evaluations of the cases are given in **Table 1**. Significant differences were found between the groups in terms of oral and dental health. The oral hygiene index (OHI) value, debris index (DI), and PBI were significantly higher in the patient group compared to the control group (p=0.045, p<0.001, and p=0.002 respectively). In addition, the patients GI was significantly higher than the control groups (p=0.001).

**Table 1:** Comparison of dental caries index values (DMFT + dmft) and gingival index and plaque index values of JIA patients (n:60) and healthy controls (n:26).

Index	Score	Patient n (%)	Control n (%)	p
OHI	**0-1.2**	7 (11.7)	4 (15.4)	0.045
	**1.3-3.0**	22 (36.7)	16 (61.5)	
	**3.1-6.0**	31 (51,6)	6 (23.1)	
DMFT	High (4.5-6.5)	26 (43.3)	10 (38.5)	0.631
	Intermediate (2.7-4.4)	14 (23.3)	6 (23.1)	
	Low (1.2-2.6)	20 (33.3)	10 (38.5)	
DI	**0-0.6**	3 (5.0)	14 (53.8)	<0.001
	**0.7-1.8**	23 (38.3)	4 (15.4)	
	**1.9-3.0**	24 (56.7)	8 (30.8)	
GI	**0.1-1.0**	10 (16.7)	14 (53.8)	0.001
	**1.1-2.0**	35 (58.3)	10 (38.5)	
	**2.1-3.0**	15 (25.0)	2 (7.7)	
PBI	**0-1.3**	23 (38.3)	19 (73.1)	0.002
	**1.4-2.7**	30 (50.0)	7 (26.9)	
	**2.8-4.0**	7 (11.7)	–	

OHI, Oral hygiene index; DMFT, Dental caries index; DI, Debris index; GI, Gingival index; PBI, Papillary bleeding index. Data for the clinical interpretation of OHI-S is as follows: 0 was considered excellent, 0.1-1.2 was considered good, 1.3-3.0 was considered insufficient, and 3.0-6.0 was considered poor. GI were evaluated on a scale of 1-3; 1: mild inflammation, minimal discoloration, no edema and no bleeding 2: moderate inflammation, redness, swelling, and bleeding on probing 3: severe inflammation, redness, edema, ulceration and tendency to spontaneous bleeding. Bold values indicate dental caries index values (DMFT + dmft), gingival index, and plaque index.

### Comparison of taxonomic weight profiles between patient and control groups

Approximately 180 different genera were detected in the oral microbiota profiles taken from patients and healthy controls. Most of the taxa were similar between the patient and control groups, and the most common genera were *Streptococcus, Neisseria, Haemophilus, Gemella, Actinomyces, Porphyromonas, Leptotrichia, Lautropia, Granulicatella, Abiotrophia, Kingella, Capnocytophaga, Aggregatibacter, Rothia*, and *Eikenella* (**Supplementary Figure 1**). *Streptococcus* and *Neisseria* were the dominant genera in both groups. According to taxonomic results, the rarest genera were *Candidatus* Protistobacter, Leptotrichiaceae, *Thiobacter, Bosea, Sutterella, Necropsobacter, Mycoplasma, Lactobacillus, Staphylococcus, Spirosoma, Microaerobacter, Brachymonas, Fretibacterium, Chryseobacterium*, and *Desulfobulbus* (**Supplementary Figure 1**).

#### Oral microbial community structure is preserved in JIA: no significant differences in alpha or beta diversity compared to healthy controls

The oral microbiota profiles of 60 patients and 26 control were compared. Alpha diversity analysis revealed that the operational taxonomic units (OTUs) observed in the patient and control groups did not differ significantly between the two groups (p = 0.65, **Supplementary Figure 2**). Similarly, in the beta diversity analysis, the OTUs observed in the patient and control groups did not have a significant difference between the two groups (p = 0.51, **Supplementary Figures 3**, **4**).

#### Differential abundance of bacterial taxa at genus, family, and order levels between JIA patients and healthy controls

The microbial composition in the patient and control groups showed significant differences at the order, family, and genus levels.

#### OTU-based differential abundance analysis reveals enrichment of periodontal pathogens in JIA and commensal taxa in controls

**Figure 1:**
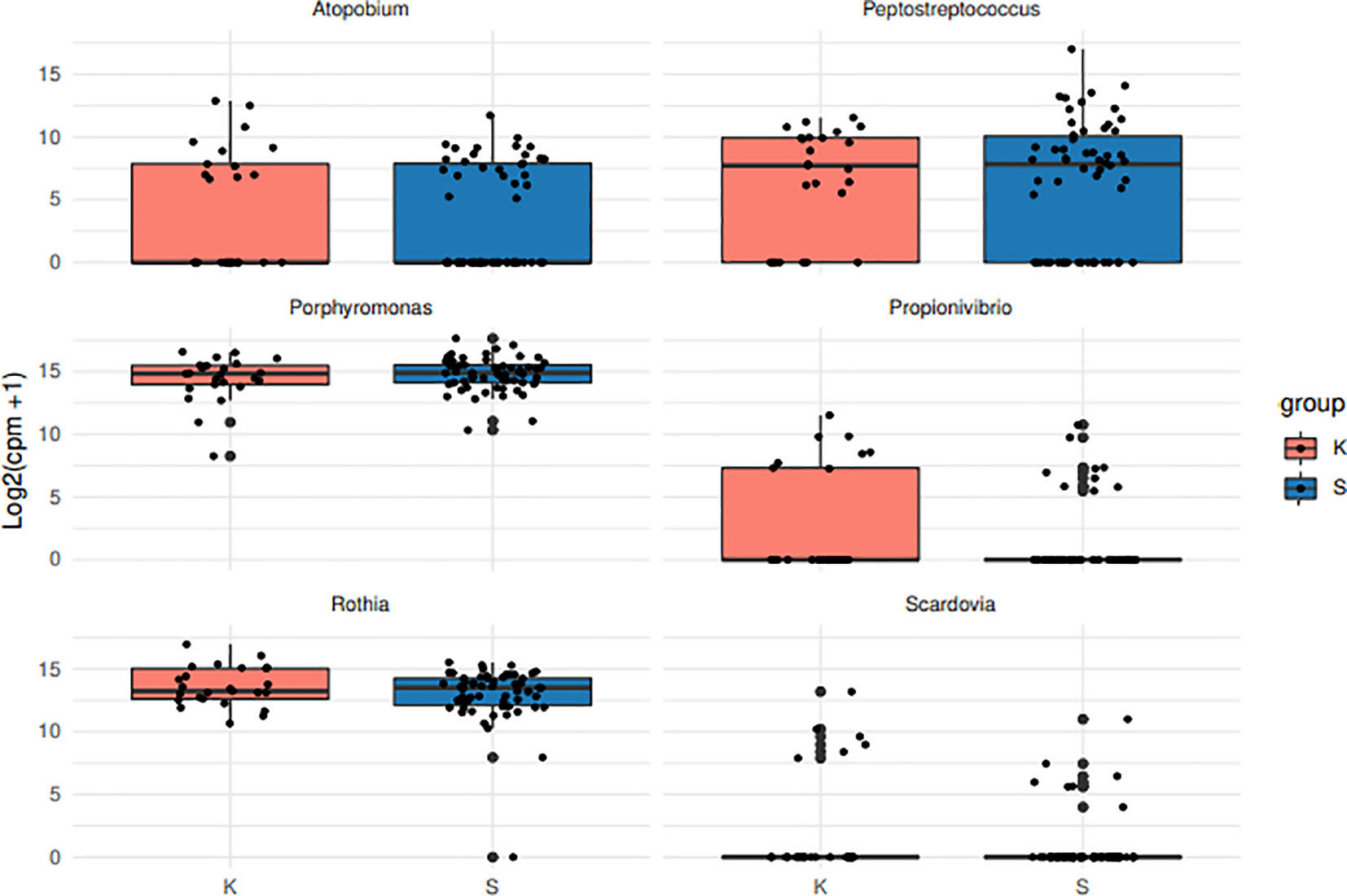
OTU-based differential abundance of bacterial genera between JIA patients and healthy controls. Subgingival plaque samples were collected from 60 children with JIA (enthesitis-related arthritis, n=31; polyarticular JIA, n=29) and 26 healthy age- and sex-matched controls. Each box plot represents the relative abundance (normalized read counts) of a single genus; the horizontal line within each box denotes the median, box edges indicate the interquartile range (IQR), and whiskers extend to 1.5×IQR. Individual data points beyond the whiskers are displayed as outliers (dots). Blue boxes (S) represent the JIA patient group; pink boxes (K) represent the healthy control group. Genera shown are those that reached statistical significance (p < 0.05, EdgeR). *Atopobium*, *Scardovia*, *Rothia*, and *Propionivibrio* were significantly enriched in controls, whereas *Porphyromonas* and *Peptostreptococcus* were significantly enriched in JIA patients.

Genus level: *Atopobium, Scardovia, Rothia*, and *Propionivibrio* were significantly higher in the control group than in the patient group (p < 0.05, gene quantity difference analysis) (**Figure 1**; **Supplementary Figure 5**). *Peptostreptococcus* and *Porphyromonas* were significantly higher in the patient group than in the control group (p < 0.05, gene quantity difference analysis) (**Figure 1**; **Supplementary Figure 5**).

**Figure 2:**
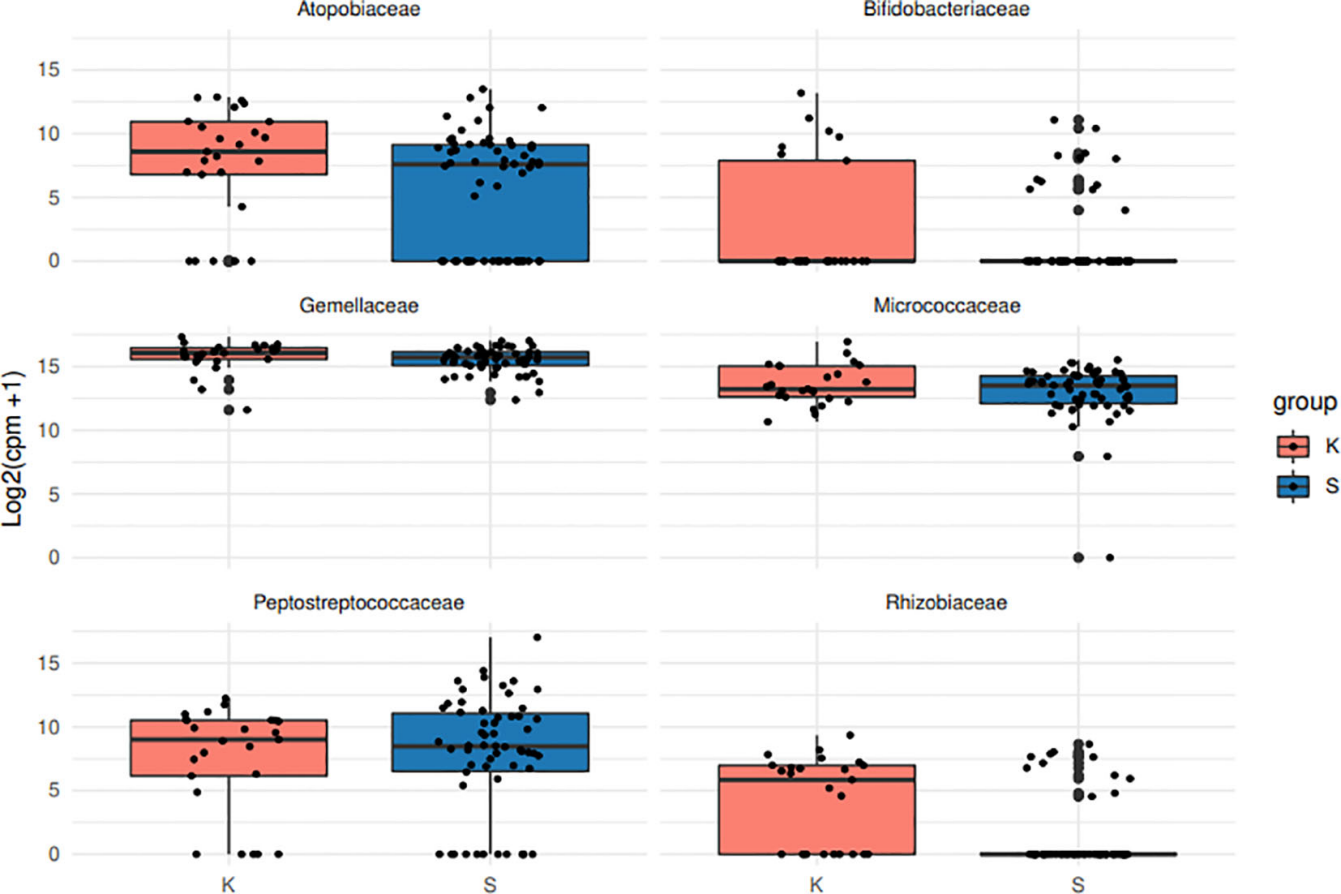
OTU-based differential abundance of bacterial families between JIA patients and healthy controls. Box plots show normalized relative abundances; the horizontal line within each box denotes the median, box edges indicate the IQR, and whiskers extend to 1.5×IQR. Outliers are shown as individual dots. Blue boxes (S) = JIA patients; pink boxes (K) = healthy controls. Only families reaching statistical significance are shown (p < 0.05, EdgeR). *Bifidobacteriaceae*, *Micrococcaceae*, *Gemellaceae*, *Atopobiaceae*, and *Rhizobiaceae* were significantly enriched in controls, whereas *Peptostreptococcaceae* was significantly enriched in JIA patients.

Family level: Bifidobacteriaceae, Micrococcaceae, Gemellaceae, Atopobiaceae, and Rhizobiaceae were significantly higher in the control group than in the patient group (p < 0.05, gene quantity difference analysis) (**Figure 2**). Peptostreptococcaceae was significantly higher in the patient group than in the control group (p < 0.05, gene quantity difference analysis) (**Figure 2**).

**Figure 3:**
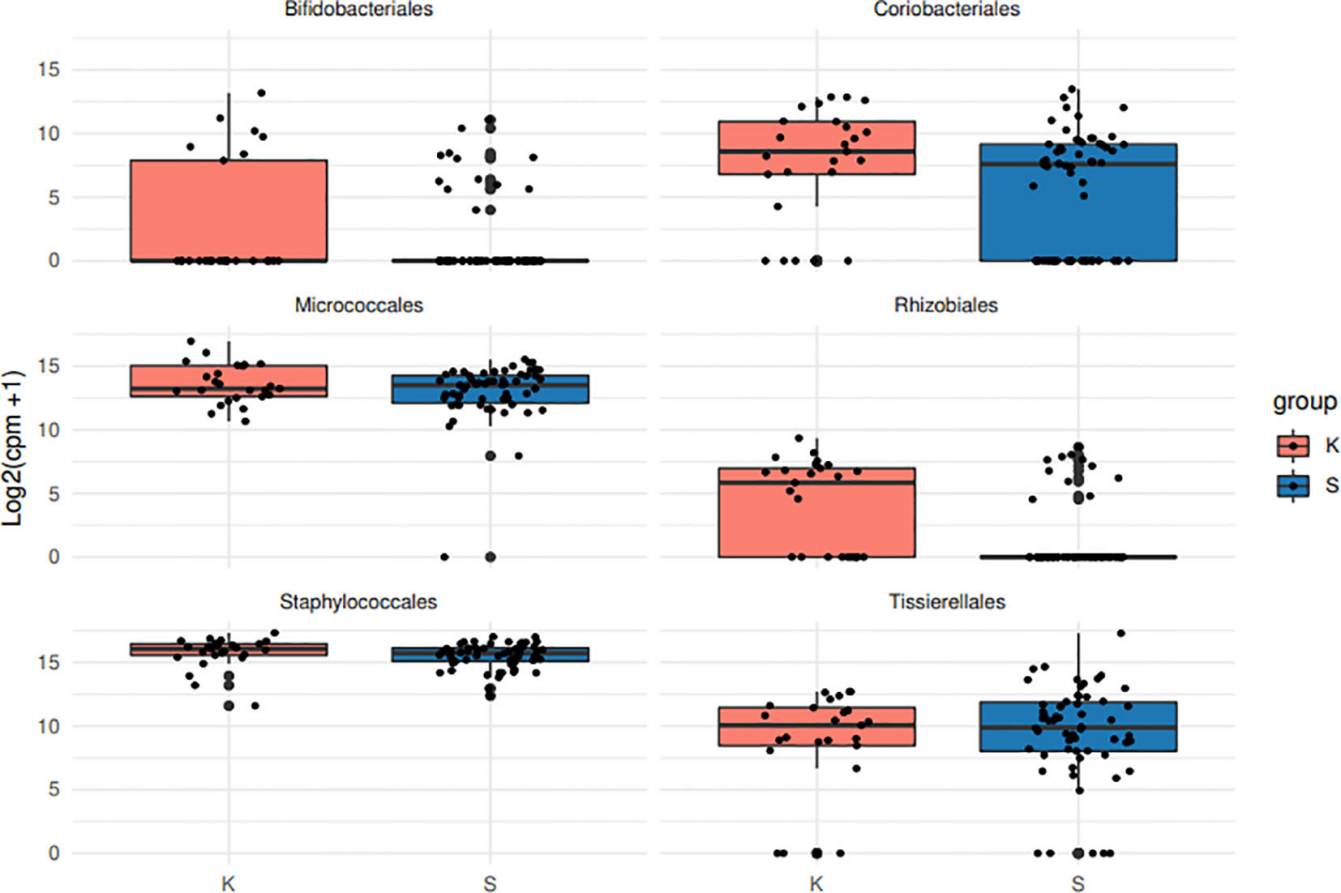
OTU-based differential abundance of bacterial orders between JIA patients and healthy controls. Box plots show normalized relative abundances; the horizontal line within each box denotes the median, box edges indicate the IQR, and whiskers extend to 1.5×IQR. Outliers are shown as individual dots. Blue boxes (S) = JIA patients; pink boxes (K) = healthy controls. Only orders reaching statistical significance are shown (p < 0.05, EdgeR). *Staphylococcales*, *Bifidobacteriales*, *Micrococcales*, *Coriobacteriales*, and *Rhizobiales* were significantly enriched in controls, whereas *Peptostreptococcales-Tissierellales* was significantly enriched in JIA patients.

Order level: Staphylococcales, Bifidobacteriales, Micrococcales, Coriobacteriales, and Rhizobiales were significantly higher in the control group than in the patient group (p < 0.05, gene quantity difference analysis) (**Figure 3**). Peptostreptococcales-Tissierellales was significantly higher in the patient group than in the control group (p < 0.05, gene quantity difference analysis) (**Figure 3**).

#### ASV-level analysis confirms enrichment of *Capnocytophaga* and *Bergeyella* in JIA, with depletion of commensal genera including *Streptococcus, Eikenella, and Propionivibrio*

**Figure 4:**
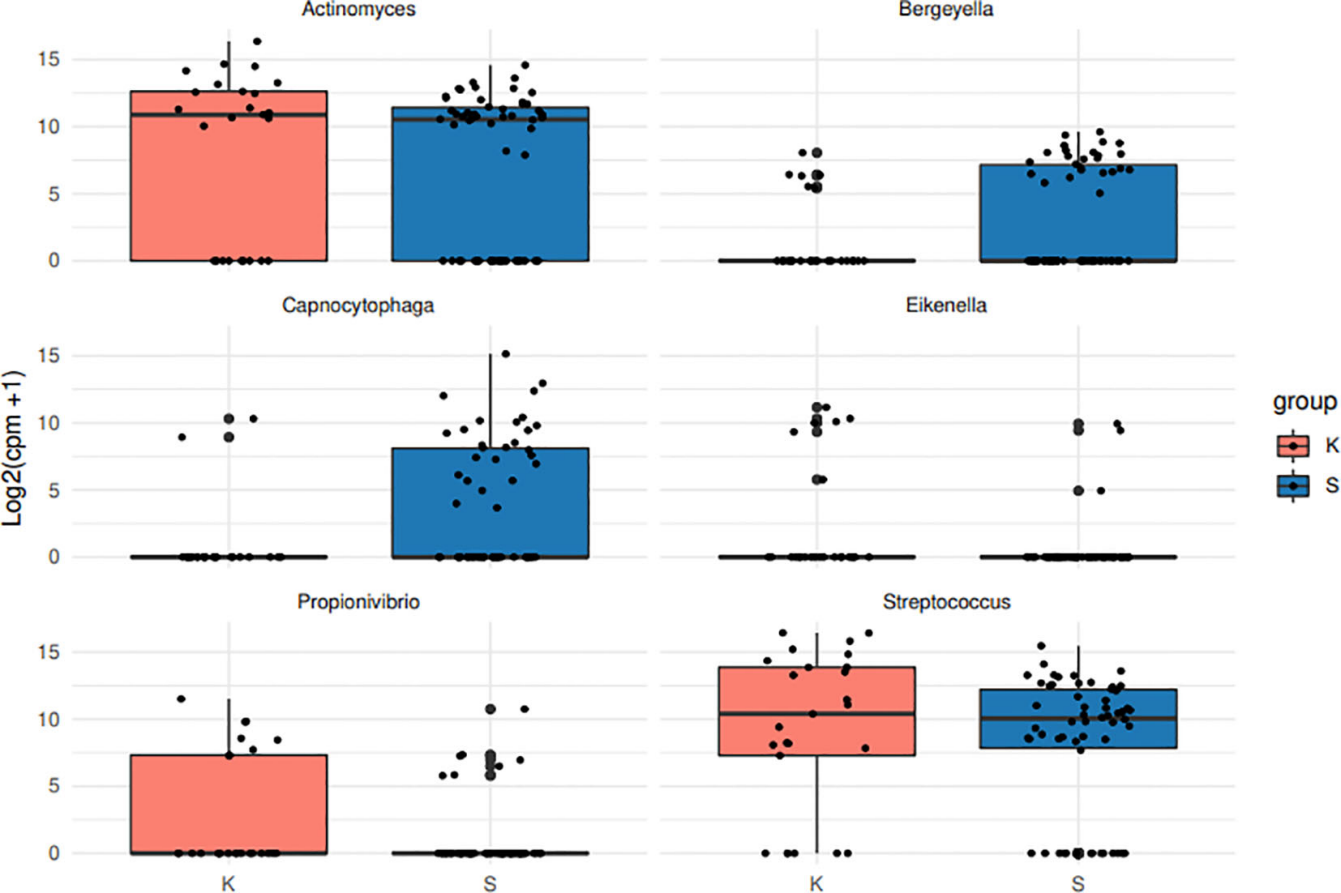
ASV-based differential abundance of bacterial genera between JIA patients and healthy controls. Box plots show normalized relative abundances at genus level; the horizontal line within each box denotes the median, box edges indicate the IQR, and whiskers extend to 1.5×IQR. Outliers are shown as individual dots. Blue boxes (S) = JIA patients; pink boxes (K) = healthy controls. Only genera reaching statistical significance are shown (p < 0.05, EdgeR). *Streptococcus*, *Eikenella*, *Actinomyces*, and *Propionivibrio* were significantly enriched in controls, whereas *Capnocytophaga* and *Bergeyella* were significantly enriched in JIA patients.

Genus level: *Streptococcus*, *Eikenella*, *Actinomyces*, and *Propionivibrio* were significantly higher in the control group than in the patient group (p < 0.05, gene quantity difference analysis) (**Figure 4**). *Capnocytophaga* and *Bergeyella* were significantly higher in the patient group than the control group (P < 0.05, gene quantity difference analysis) (**Figure 4**).

#### Comparison of microbial abundance between ERA and polyarticular groups

Based on the analyses performed, no significant difference was found in microbial abundance between the polyarticular (POL) and enthesitis-related arthritis (ERA) groups. Analyses at the order level (Campylobacterales, Tissierellales), family level (Campylobacteraceae, Porphyromonadaceae), and genus level (*Campylobacter, Johnsonella, Porphyromonas*, and *Scardovia*) reveal that median values and data distributions (on the Log2(CPM + 1) scale) remain closely aligned across both groups (**Supplementary Figures 6**–**8**).

## Discussion

This study aimed to investigate the potential association between the oral microbiome and inflammatory arthritis by comparing the subgingival plaque microbiota of patients with juvenile idiopathic arthritis (JIA) with that of a healthy control group. Our findings revealed that children with JIA had higher markers of gingival inflammation compared to healthy controls and exhibited changes in the distribution of different bacterial taxa. In particular, the increased abundance of periodontal pathogens such as *Porphyromonas* and *Peptostreptococcus* in the JIA group suggests that oral microbial dysbiosis may be linked to inflammatory processes involved in the pathogenesis of inflammatory arthritis.

The present study demonstrated that gingival inflammation was significantly higher in children with JIA compared to healthy controls. This finding is consistent with the results of other studies investigating JIA and oral health (Frid et al., 2020; Grevich et al., 2019; Leksell et al., 2008; Santos et al., 2015). In our study, it was determined that children with JIA had an increased papillary bleeding index (PBI), a marker of gingival inflammation. Similarly, other studies have reported high PBI values in children with JIA (Frid et al., 2020; Greenstein et al., 1981; Grevich et al., 2019; Tanner et al., 2007; Teles et al., 2010). Frid et al. evaluated OHI-S and PBI findings in 59 JIA patients and identified significantly higher inflammation scores compared to a control group of healthy children (Frid et al., 2020). Grevich, on the other hand, compared 81 JIA patients with a control group consisting of 11 healthy and 62 dental patients and similarly identified high PBI values in children with JIA (Grevich et al., 2019). It is known that PBI is associated with the risk of progression to periodontitis, the extent of inflammatory infiltration in gingival tissue, and the levels of pro-inflammatory mediators in the gingiva, such as interleukin-1β, interleukin-8, and matrix metalloproteinases, and is considered a specific clinical indicator of gingival inflammation (Grevich et al., 2019; Teles et al., 2010). The co-occurrence of periodontitis and inflammatory arthritis suggests the presence of a common immune dysregulation in both tissues (Cole and Kuhn, 2025; Holers et al., 2018; Tang et al., 2023).

The Simplified Oral Hygiene Index (OHI-S) is another parameter shown to be elevated in patients with JIA; this finding reflects gingival inflammation and bacterial proliferation (Frid et al., 2020; Reichert et al., 2006). In the present study, elevated OHI-S values were observed in children with JIA. Similarly, Frid and colleagues also reported high OHI-S values in JIA patients (Frid et al., 2020). The Gingival Index (GI) is another parameter that assesses gingival inflammation, and in this study, it was found to be significantly higher in the JIA group compared to controls. Grevich also found higher GI values in children with JIA (Grevich et al., 2019). Pathogenic bacterial colonization associated with chronic inflammation in JIA can be explained by the oral microbial dysbiosis observed in these patients (Cole and Kuhn, 2025). In the majority of studies in the literature, microbial dysbiosis has been found to be associated with the abundance of taxa linked to chronic inflammation in JIA; furthermore, it is thought that this dysbiosis may be driven more by strong community effects rather than the actions of individual microbes (Frid et al., 2020; Grevich et al., 2019; Cole and Kuhn, 2025).

The insignificance of alpha or beta diversity analyses between the patient and control groups in this study is consistent with previous studies (Frid et al., 2020; Grevich et al., 2019).This observation suggests that changes in the microbiota in JIA may be driven not by the proliferation of a single pathogenic microorganism, but by broader shifts in microbial composition and ecological balance. Periodontitis is directly associated with bacterial plaque accumulation resulting from poor oral hygiene, which involves pathogens such as *Porphyromonas gingivalis*, *Prevotella*, *Tannerella, Eikenella*, and *Fusobacterium* (Siddiqui et al., 2023).

The present study revealed that *Porphyromonas* and Peptostreptococcales-Tissierellales are abundant in the microbiota of children with JIA. Similarly, Grevich et al. demonstrated an enrichment of *Porphyromonas* and *Rothia* in subgingival plaque samples collected from patients with polyarticular JIA (Grevich et al., 2019). Stoll and colleagues likewise demonstrated the abundance of *Porphyromonas* in subgingival plaque and Rothia in saliva samples from patients with juvenile spondyloarthritis (Stoll et al., 2022). Serologically, it has been shown that patients with JIA have higher levels of antibodies against *Prevotella intermedia* (IgG), *Prevotella oralis* (IgA/IgG), and *Porphyromonas gingivalis* (IgG) compared to healthy controls (Romero-Sanchez et al., 2017; Stoll et al., 2020; Zekre et al., 2022). Additionally, it has been reported that patients with positive *Porphyromonas gingivalis*-IgG1 antibodies are more likely to be ACPA (anti-citrullinated protein antibodies) positive (Romero-Sanchez et al., 2017). These findings suggest that immune responses directed against oral (periodontal) pathogenic bacteria may contribute to the pathogenesis of inflammatory arthritis.

It has been demonstrated that periodontopathogenic bacteria such as *Porphyromonas gingivalis* contribute to the formation of anti-citrullinated protein antibodies (ACPA) in RA, and that associated dysbiosis and periodontitis are linked to an increase in disease severity (Frid et al., 2020; Scher and Abramson, 2011). *Porphyromonas gingivalis* is the only known prokaryote encoding the peptidylarginine deiminase (PAD) enzyme, which converts arginine residues into citrulline and produces neoepitopes that can increase the production of ACPA. ACPA are currently the most specific biomarkers for RA and are known to be associated with a poor prognosis. A widely held hypothesis suggests that *Porphyromonas gingivalis* and the resulting humoral immune response serve as an antigenic source for the formation of ACPAs and subsequent progression to RA. Studies have shown that PAD derived from *Porphyromonas gingivalis* can citrullinate human fibrinogen and alpha-enolase isoforms, and that human alpha-enolase can also induce autoimmunity and inflammatory arthritis in DR4-transgenic mice (Wegner et al., 2010). In addition, it has been reported that periodontitis treatment reduces the levels of circulating ACPA and anti-*Porphyromonas gingivalis* antibodies (Okada et al., 2013). In particular, this demonstrates a strong association between the presence of *Porphyromona*s and the development of inflammatory arthritis in patients with periodontitis. It is conceivable that this well-defined mechanism in RA pathogenesis (the PAD/ACPA pathway) could indicate an autoimmune trigger associated with the *Porphyromonas* enrichment we observed in children with JIA. These findings suggest that *Porphyromonas* may be a consistent finding in oral dysbiosis associated with JIA.

The present study has demonstrated that *Peptostreptococcus* (Peptostreptococcales-Tissierellales) are more abundant in the JIA group. The association of *Porphyromonas* species with JIA is consistent with findings from previous studies. In contrast, the increased abundance of Peptostreptococcales-Tissierellales has been reported less frequently in the literature and may represent a new observation contributing to the understanding of microbial changes associated with JIA. However, one study noted that in elderly patients in whom these bacteria were abundantly detected, bone metabolism was impaired, creating a risk for hip fractures (Roselló-Añón et al., 2023). Specifically, certain components of these bacteria (such as lipopolysaccharides and flagellin) have been shown to enhance the immune response by inducing cytokine synthesis, leading to increased levels of numerous cytokines associated with inflammatory disease (IL-1, IL-6, IL-17, TNF-alpha, and RANKL) (Roselló-Añón et al., 2023).

Frid et al. found abundance of the *Capnocytophaga oral taxon 864, Leptotrichia species (oral taxon 417*), *TM7-G1, Rothia, Veillonella*, and *Mogibacterium* in JIA patients (Frid et al., 2020). The prominence of different taxa in their study can largely be attributed to differences in sampling sites. While they evaluated the salivary microbiome, our study focused on subgingival plaque samples, which are the primary site of periodontal disease; therefore, it is expected that the two studies reflect different microbial niches. The study by Grevich et al. focused on subgingival plaque samples and patients with polyarticular JIA (Grevich et al., 2019). In our study, patients with polyarticular JIA and enthesitis-related arthritis were evaluated together, and no significant microbial differences were found between these subgroups. Additionally, differences exist in other taxonomic groups, which may be attributed to variations in sampling sites, patient populations, treatment status, oral hygiene, geographic and environmental factors, and the analytical methods used (**Table 2**).

**Table 2:** Comparison of microbiota profiles of healthy controls and JIA patients with other studies.

	Present study	Grevich et al. (2019)	Frid et al. (2020)
Patients	*Porphyromonas* *Peptostreptococcus* (P.Tissierellales)	*Porphyromonas* *Rothia* *Streptococcus* *Proteobacter* *Leptotrichia*	*Capnocytophaga spp* *Rothia aeria* *Alloprevotella spp* *Solobacterium* *Prevotella nanceiensis* *Mog,bacterium diversum* *Veilonella* *TM7* *Leptotrichia* spp *OT417*
Healthy controls	*Atopobium* (Actinobacteria-Atopobiacea) *Scardovia* (Actinobacter-Bifidobacter) *Rothia* (Actinobacter-Micrococcea) *Propionivibria* (Proteobacteria-Rhodocyclacea)	*Actinomyces* (Actinobacteria-Actinomycetaceae) *Corynebacteria* (Actinobacteria-Corynebacteriaceae) *Prevotella* (Bacteroidetes-Prevotellaceae)	*Rothia* *Bergeyella* *Prevotella* *Leptotrichia* *Hemophylus*

In the study by Miranda et al., 23% of the JIA group and 4% of the control group exhibited signs of periodontal disease (Miranda et al., 2005). Although lower levels of *Fusobacterium nucleatum, Campylobacter rectus, Peptostreptococcus micros*, and *Streptococcus intermedius* were detected in the subgingival plaque of JIA patients compared to the control group, no difference was found in *Porphyromonas gingivalis* or *Prevotella intermedius*. Although this study’s methodology limits its interpretability due to the absence of quantitative measurements or specific tests, it suggests the presence of an oral dysbiosis distinct from that of the healthy group (Miranda et al., 2005).

Overall, our findings support the concept that JIA may be associated with microbial dysbiosis rather than the presence of a single specific pathogen. Similarly, current evidence suggests that JIA is associated with changes in the microbial composition and an increase in the abundance of taxa linked to chronic inflammation (Chriswell and Kuhn, 2020; Cole and Kuhn, 2025; Frid et al., 2020; Grevich et al., 2019; Lange et al., 2016; Siddiqui et al., 2023). However, it remains unclear whether these changes in the oral microbiota are a causal factor in disease development or secondary to ongoing inflammatory processes (Cole and Kuhn, 2025).

The 16S rRNA V3–V4 sequencing approach used in this study allows for reliable characterization of the microbiota at the genus level; however, its resolution at the species level is inherently limited. Consequently, it is not possible to definitively identify specific pathogenic species such as *Porphyromonas gingivalis*. This methodological limitation should be taken into account when interpreting the findings. Future studies utilizing higher-resolution techniques, such as shotgun metagenomic sequencing, may provide more detailed information regarding the species-level composition and functional potential of the oral microbiome.

This study has several additional limitations. All included patients were receiving immunomodulatory therapy, and the potential effect of these treatments on the composition of the oral microbiota is not fully understood. Although antibiotic use was restricted prior to sampling, oral hygiene practices were not standardized. While the study was initially designed to compare different JIA subtypes separately, the absence of significant differences among the subgroups led to their consolidation into a single JIA cohort and resulted in a larger patient group relative to the controls. This imbalance may have affected statistical power and the sensitivity of the comparisons.

The findings of this study should be interpreted in the context of various potential confounding factors. Oral hygiene practices, dietary habits, and socioeconomic status are well-known determinants of oral microbiota composition and gingivitis and may have influenced the observed associations. Since these variables were not comprehensively assessed, it cannot be definitively determined whether the identified microbial differences can be attributed solely to disease-related mechanisms. Future studies incorporating a detailed evaluation and appropriate control of these factors are needed to better elucidate the relationship between the oral microbiota and JIA.

## Conclusion

In children with JIA, differences in the abundance of various oral bacterial taxa (*Porphyromonas, Peptostreptococcus*) were observed compared to healthy controls. However, given the cross-sectional nature of this study, these findings should be interpreted as an association rather than evidence of causality. While the observed changes may indicate a potential interaction between the oral microbiota and local inflammatory processes, their contribution to the onset or severity of the disease remains unclear. Large-scale, longitudinal, and mechanistic studies are needed in the future to clarify the underlying temporal and causal relationships and to determine whether the oral microbiome can serve as a biomarker or therapeutic target in JIA.

## Data Availability

The datasets presented in this study can be found in online repositories. The names of the repository/repositories and accession number(s) can be found in the article/**Supplementary Material**.
