# Supplementary material for: Oral health status and subgingival microbiota in children with juvenile idiopathic arthritis
Source: Front Cell Infect Microbiol. 2026 Jun 26;16:1831655. doi: 10.3389/fcimb.2026.1831655 (PMC13350034; doi:10.3389/fcimb.2026.1831655)
Supplement: Supplementary file 1 [file DataSheet1.pdf]

Supplementary material:

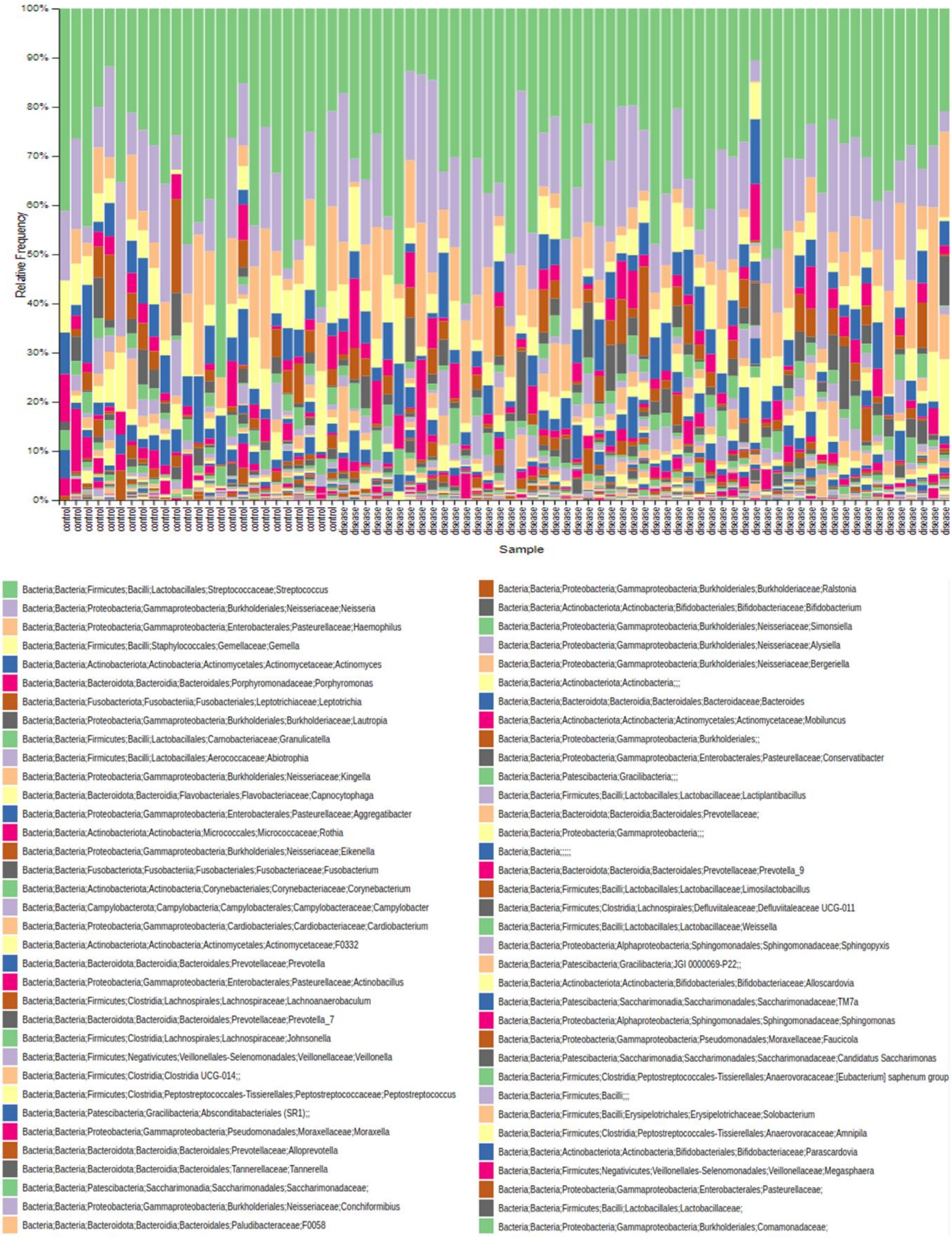

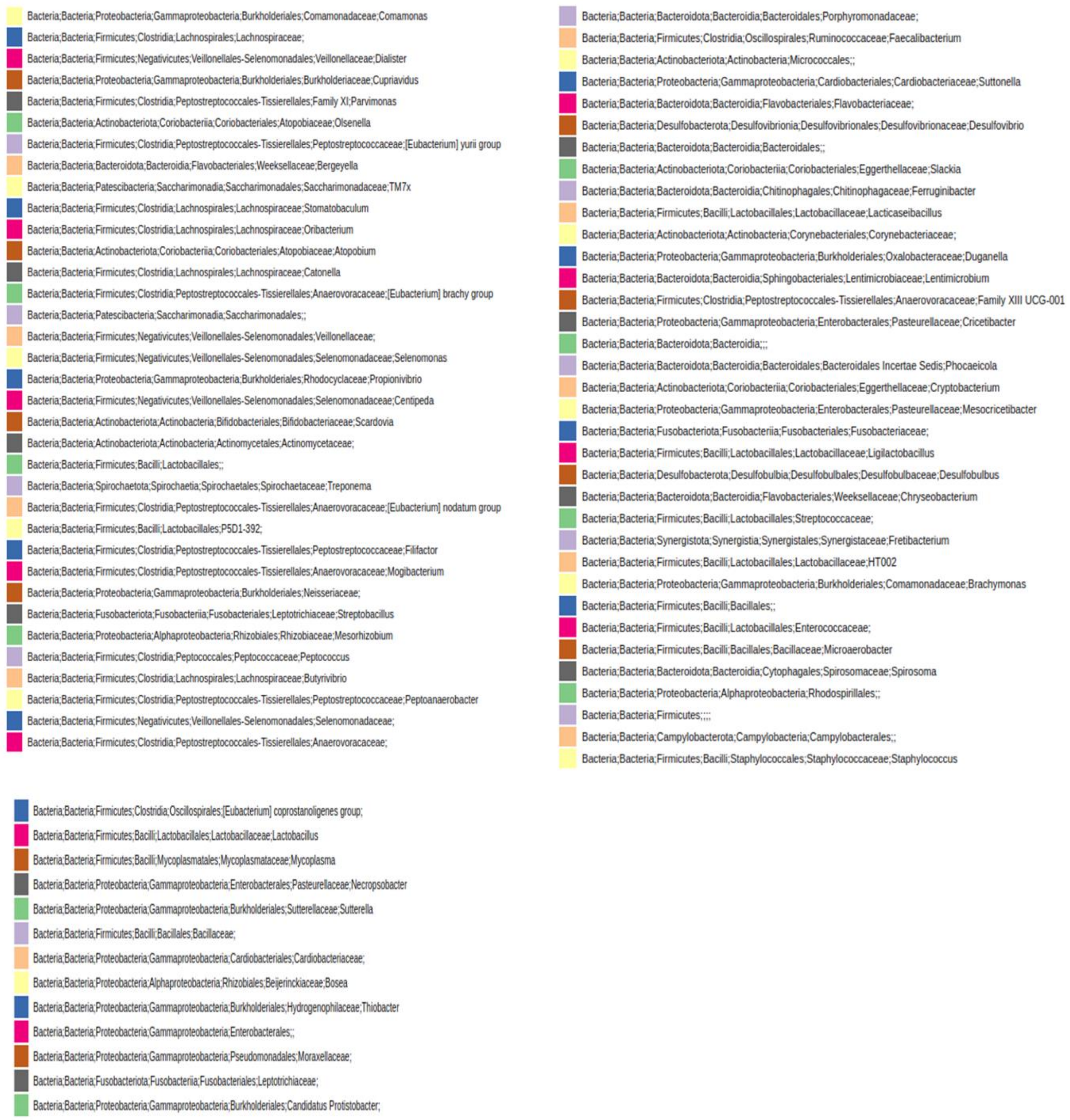

Supplementary Figure 1. Comparison of profiles of oral microbial composition in patient and control groups. Graph of taxonomic profiles of patient and control groups at the genus level.

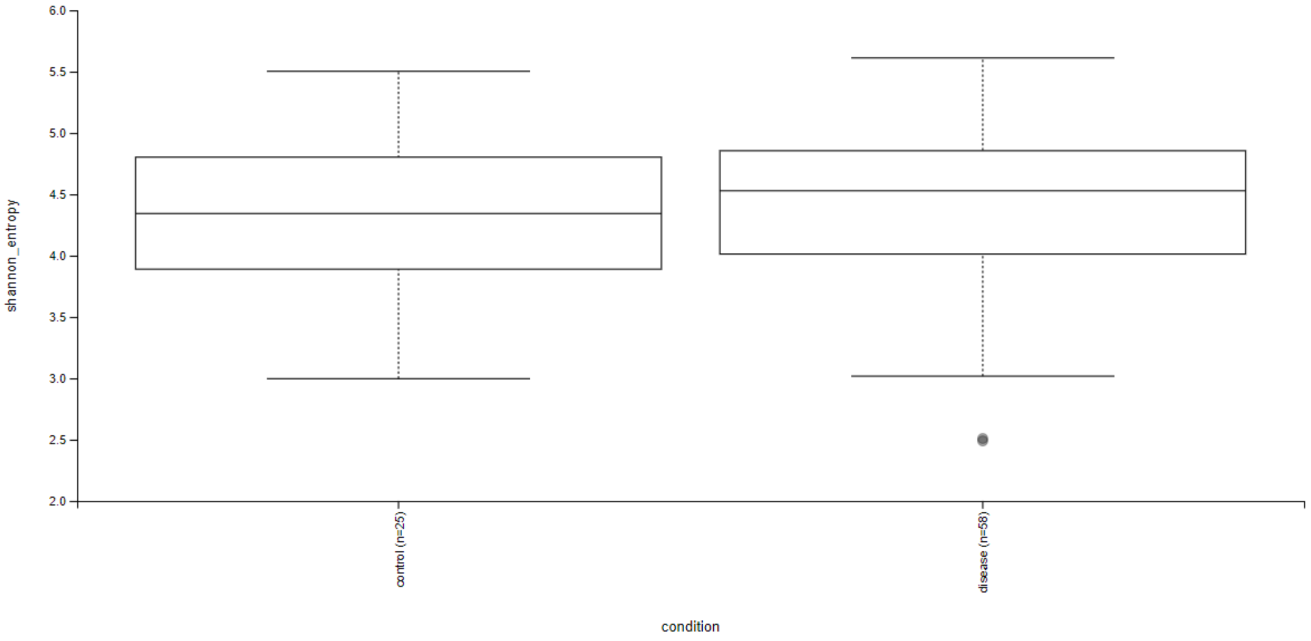

Supplementary Figure 2. Alpha-diversity of the oral microbiota (Shannon index) in the patient and control group. Box-mustache graph is shown for the patient and control groups. The line inside each box represents the median value. Outliers are shown as dots.

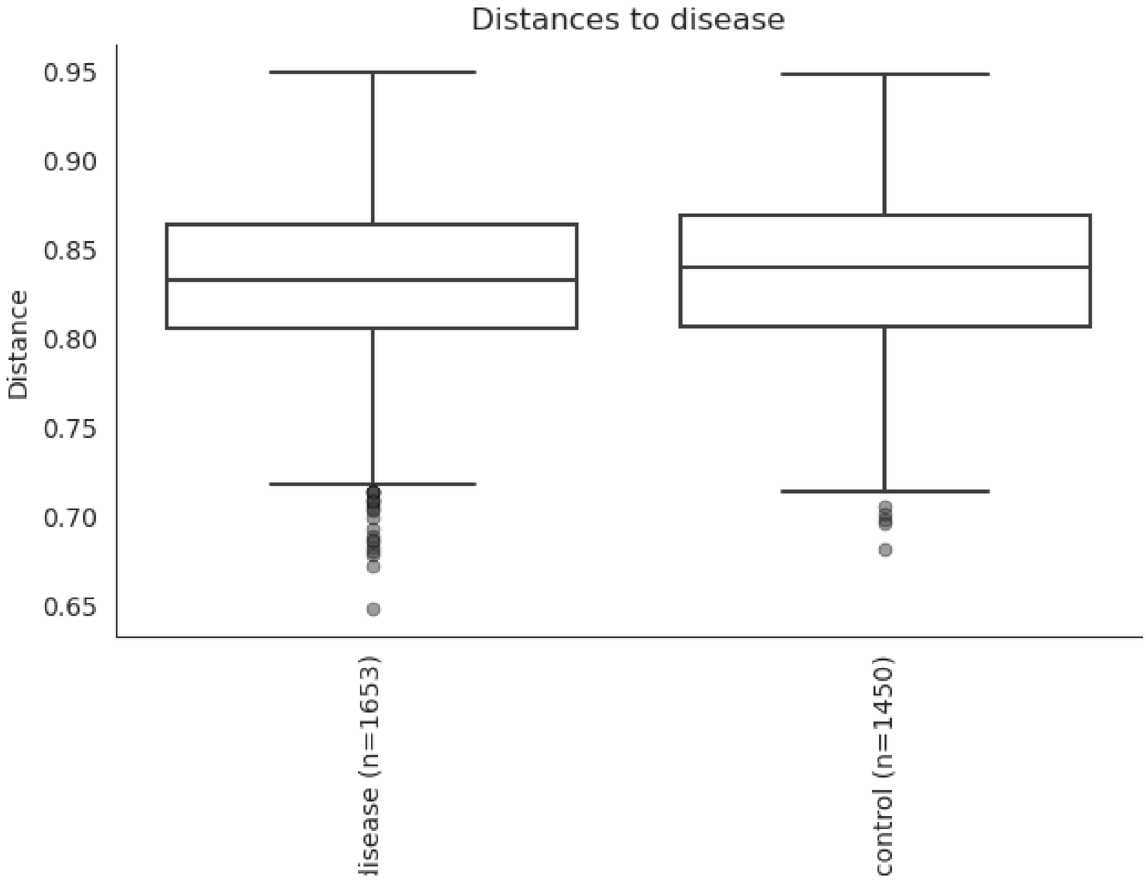

Supplementary Figure 3. Beta-diversity of the oral microbiota in the patient and control group (Jaccard distance matrix). The box-mustache graph of the Jaccard distance matrix for the patient and control groups is shown. The line inside each box represents the median value. Outliers are shown as dots.

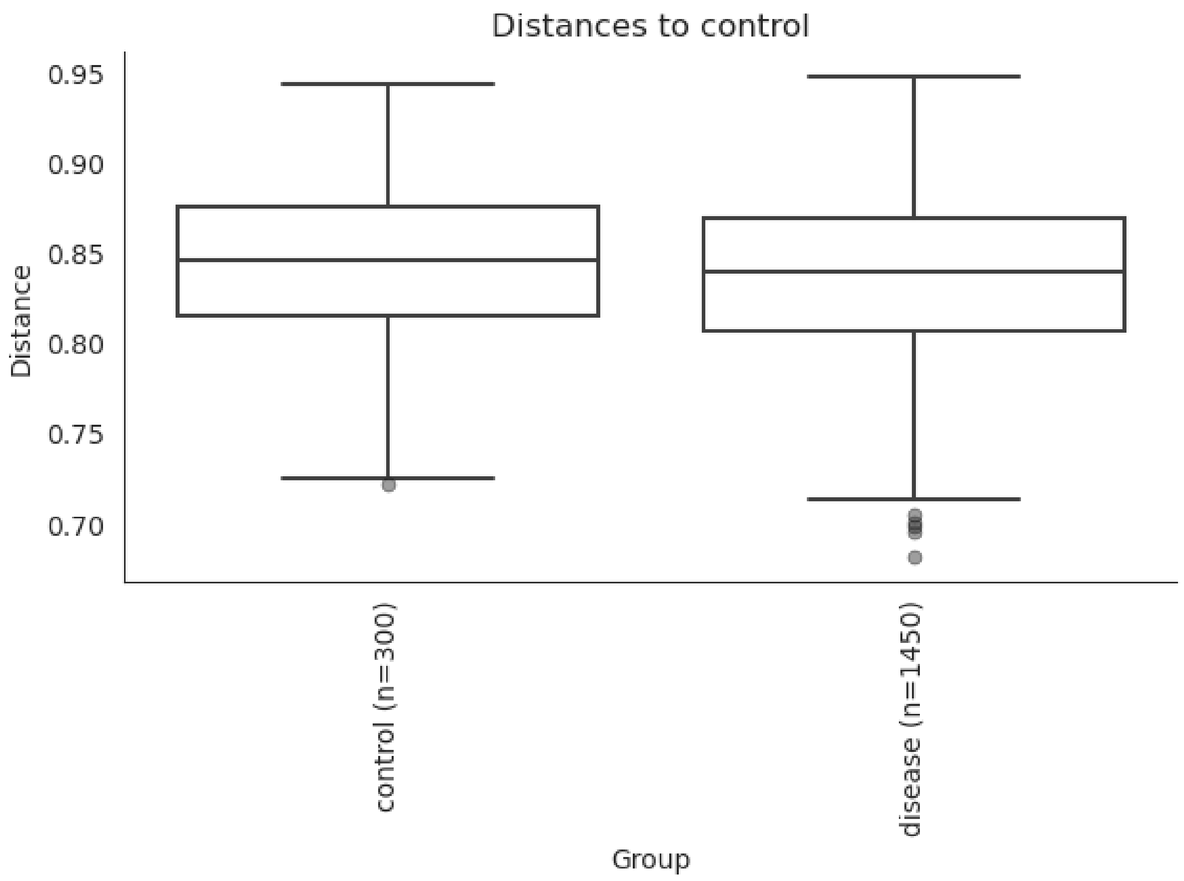

Supplementary Figure 4. Beta diversity index (Jaccard distance matrix). The box-mustache graph of the jaccard distance matrix for the patient and control groups is shown. The line inside each box represents the median value. Outliers are shown as dots.

OTU.ID	logFC (JIA vs. control)	logCPM	*P-value*	FDR
Bacteria; Bacteria; Actinobacteriota; Actinobacteria; Bifidobacteriales; Bifidobacteriaceae; *Scardovia*	-4.200	8.6273	0.0006	0.0195
Bacteria; Bacteria; Actinobacteriota; Coriobacteria; Coriobacteriales; Atopobiaceae; *Atopobium*	-2.7657	9.0476	0.0004	0.0195
Bacteria; Bacteria; Firmicutes; Clostridia; Peptostreptococcales-Tissierellales; Peptostreptococcaceae; *Peptostreptococcus*	2.0731	11.1328	0.0145	0.2721
Bacteria; Bacteria; Actinobacteriota; Actinobacteria ; Micrococcales; Micrococcaceae; *Rothia*	-0.7727	14.1391	0.0221	0.3095
Bacteria; Bacteria; Proteobacteria; Gammaproteobacteria; Burkholderiales; Rhodocyclaceae; *Propionivibrio*	-2.1321	6.6507	0.0289	0.3243
Bacteria; Bacteria; Bacteroidota; Bacteroidia; Bacteroidales; Porphyromonadaceae; *Porphyromonas*	0.7024	15.3827	0.0366	0.3416

Supplementary Figure 5. FDR and P values of bacterial genera that differ significantly between the two groups.

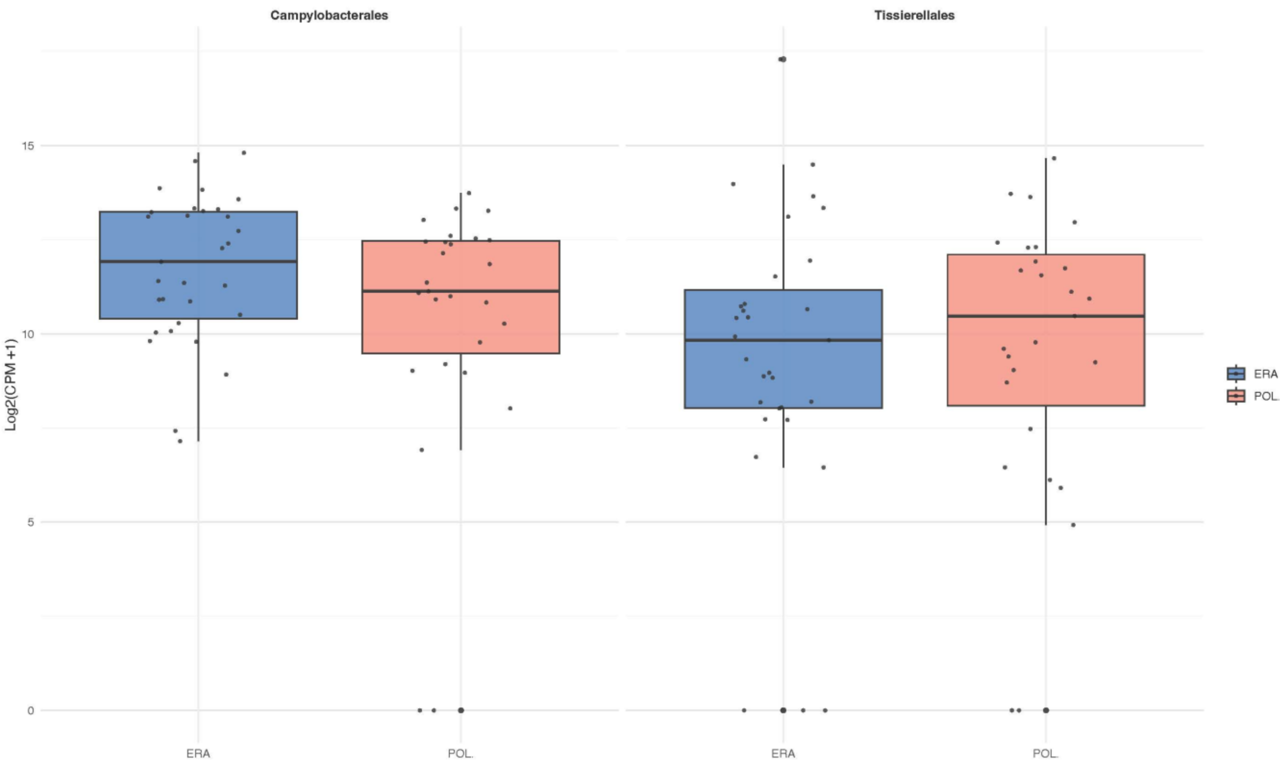

Supplementary Figure 6. Comparison of bacterial orders, including Campylobacterales and Tissierellales, between the POL and ERA groups (calculated using the QIIME 2 pipeline). No significant differences were found between the groups. The line inside each box represents the median value, and outliers are shown as dots. Pink boxes (POL) represent the polyarticular group, and blue boxes (ERA) represent the enthesitis-related arthritis group.

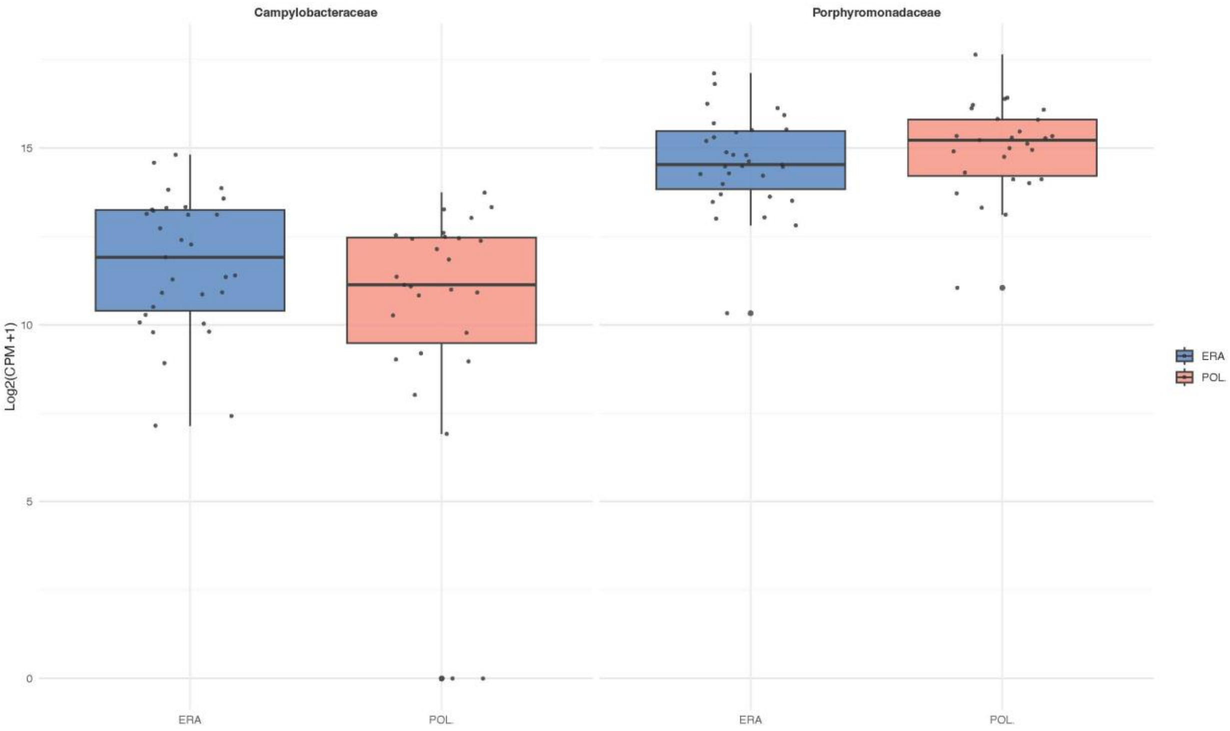

Supplementary Figure 7. Comparison of bacterial families, including Campylobacteraceae and Porphyromonadaceae, between the POL and ERA groups (calculated using the QIIME 2 pipeline). No significant differences were found between the groups. The line inside each box represents the median value, and outliers are shown as dots. Pink boxes (POL) represent the polyarticular group, and blue boxes (ERA) represent the enthesitis-related arthritis group.

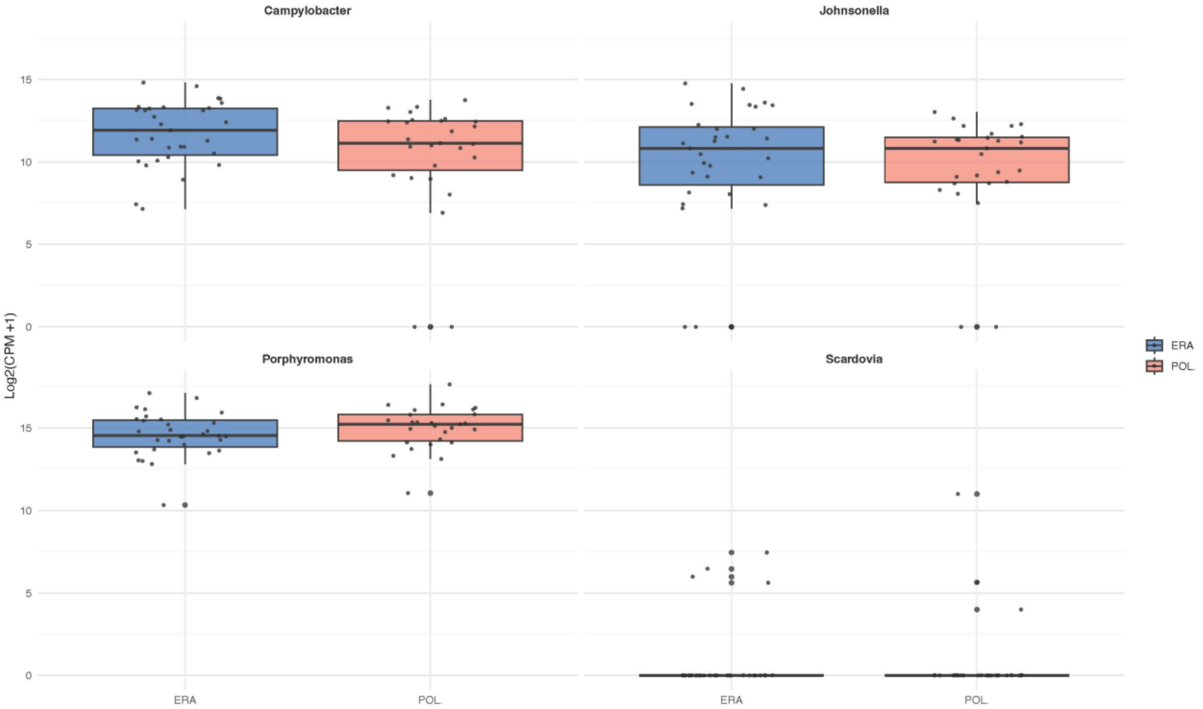

Supplementary Figure 8. Comparison of bacterial genera, including *Campylobacter*, *Johnsonella*, *Porphyromonas* and *Scardovia* between the POL and ERA groups (calculated using the QIIME 2 pipeline). No significant differences were found between the groups. The line inside each box represents the median value, and outliers are shown as dots. Pink boxes (POL) represent the polyarticular group, and blue boxes (ERA) represent the enthesitis-related arthritis group.
